# Editorial: The role of sex in cardiac arrhythmias and sudden cardiac death

**DOI:** 10.3389/fcvm.2023.1158376

**Published:** 2023-03-03

**Authors:** Matias E. Pollevick, Elaine Y. Wan

**Affiliations:** ^1^Division of Cardiology, Columbia University Vagelos College of Physicians and Surgeons, Columbia University, New York, NY, United States; ^2^Department of Medicine, Columbia University Vagelos College of Physicians and Surgeons, Columbia University, New York, NY, United States

**Keywords:** atrial arrhythmia, ventricular arrhythmia, sex, sudden cardiac death, risk stratification, cardiovascular disease (CVD), ventricular fibrillation (VF), ventricular tachycardia (VT)

## Introduction

Sudden cardiac death (SCD) when it occurs in the public eye to professional athletes or celebrities, garners media attention and public health interest to improve understanding of its causes and prevention in other athletes. The aftermath is then to stratify the survivor's risk return to play. Sudden cardiac death, just like other cardiac arrhythmias, has been recognized that its epidemiology, and response to treatment differ significantly between men and women. There are known sex differences in cardiac electrophysiology and clinical outcomes. Women have higher resting heart rates and longer baseline electrocardiographic QT intervals which affect arrhythmic risk. Yet, despite all this understanding there is a lack of guideline directed screening or risk assessment for men and women in competitive sports.

## Review of sudden cardiac death

Sudden cardiac death (SCD) in the United States remains a major driver of mortality. Estimates of prevalence site as many as 325,000 deaths annually and as many as 1,000 deaths per day (1). SCD accounts for as many as 50% of all cardiovascular deaths, with as many as half of those deaths marking the sentinel event in manifestation of CVD (2). Most causes of SCD are driven by ischemic heart disease, however, recently the incidence of ischemic driven SCD is decreasing, whereas the role of cardiomyopathies tightly linked to fibrosis and left ventricular hypertrophy are increasing (3). In addition, Haukilahti et al. (3) showed that although the annual rate of SCD in women is about half of that in men, the rate of SCD in men has been declining more rapidly than in women. Interestingly, during subgroup analysis, these investigators found primary myocardial fibrosis to have a higher prevalence of causing non-ischemic SCD among women as compared to men. However, there continues to be an under recognition of this increased risk for women as compared to men in much of the scientific literature.

## Screening for arrhythmias

Athletes present a special population in SCD from arrhythmias due to the physical and emotional stress that they place on their cardiovascular support system. In their study of National Basketball Association (NBA) athletes, Waase et al. (4) discuss the accuracy of existing athlete-specific electrocardiographic (ECG) criteria used as part of the NBA's required yearly health screening. After analyzing ECG data, the authors concluded that abnormal classification rates were higher among NBA athletes than other athletic sports. Even after analyzing the data through three different classification systems. T-wave inversions were the most common ECG abnormality.

On January 2nd, 2023, professional football player Damar Hamlin suffered a direct blow to his chest from the head of an opposing player, causing a sudden collapse view by television viewers nationwide. He suffered SCD and required advanced cardiac resuscitation on the field while encircled by teammates—an event that shocked the sporting world and general media. Thankfully, after appropriate and swift therapy he is recovering well. It is thought that the event leading to his SCD was commotio cordis, ventricular fibrillation (VF) triggered by a non-penetrating blow to the chest which triggered a ventricular extra stimulus during the ventricular repolarization period, a “R on t” phenomenon inciting ventricular fibrillation. Although the exact incidence of commotio cordis is unknown, it is one of the most frequent causes of SCD in young athletes. In their 2010 review, Maron and Estes (5) found the most common sports where commotio cordis occurred were baseball and softball, followed by hockey and then football. Data on commotio cordis are limited, but insight regarding the intricacies and complications of commotio cordis may be gleaned from case reports such as that by Westreich et al. (6). They discuss the case of 35-year-old soccer player who had a blow to the chest, and was subsequently found to have sustained ventricular tachycardia (VT) during electrophysiology study after having an episode of regular wide complex tachycardia on the field 9 months after his initial commotio cordis event. Out of all the variables that must align to cause these rare events, the authors discuss that only blows occurring during the 20–40 ms window as the T wave is trending upwards can cause VF. The authors explain that adenosine triphosphate–sensitive potassium channels are activated by force to the chest causing problems with repolarization, ultimately leading to arrhythmia. Although the event is uncommon, it is often deadly.

How can we prevent these events in athletes? One aspect of prevention is related to screening, while the other may be related to increased protection. There is currently no international recommendation from professional societies regarding ubiquitous cardiac screening, specifically screening with electrocardiogram (ECG) or transthoracic echocardiography (TTE), and/or magnetic resonance imaging for athletes. Corrado et al. demonstrated that after following over 30,000 Italian athletes for 25 years, they were able to successfully identify athletes with hypertrophic cardiomyopathy in a manner that was adequately sensitive and cost-effective (7). They compared their rate of detection to prevalence in other countries and found the rates to be comparable given the populations (0.07% in Italy vs. 0.1% in the US by TTE). It is difficult to extrapolate cost and feasibility from studies done nearly 20 years ago, but screening with 12-lead ECG may still be the best way to identify those who may be predisposed to SCD due to structural heart disease, channelopathies or abnormalities in cardiac conduction. Commotio cordis may just be the event that exposed an underlying structural cardiac problem. With more widespread screening programs, athletes with these underlying anomalies would be evaluated and treated accordingly. However, Bickel et al. review some of the limitations of widespread screening, including a high number needed to screen (2,000), lack of cost-effectiveness from adding EKG to the history, and need to focus on secondary prevention instead of screening (8). The authors discuss the need to ensure AEDs are readily available at all major sporting events, as neurologically intact survival rates were found to be 3.9-fold higher in patients where an AED was onsite. Lastly, they discuss the differences between the European and American guidelines, the later deferring the use of 12-lead ECG screening given the lack of robust data and financial infeasibility. More debate is yet to come.

But even after surviving SCD, what are the requirements for return to play? A court ruling in the case of SCD in a collegiate basketball player, *Knapp* v. *Northwestern University*, Maron et al. (9) the legal decision of the United States Seventh District Court upheld the decision of team physicians at Northwestern University to prohibit an athlete from participating in competitive sports given his history of VF, a high risk medical conditions. The ruling cites that both the power and the responsibility fall with physicians to ensure that young athletes are safe to partake in competitive sports. The courts' arguments for allowing the university to prohibit the student from playing relied on three key factors, namely the decision of the team's physician, opinions from appropriate specialists, and consensus guidelines, such as those established at several Bethesda Conferences. Society, law, and medicine have all changed drastically since 1996 when this precedent was made and they are, by nature, ever evolving disciplines. The need for ongoing revision and evaluation of current guidelines based upon the evolution of scientific advancement if highlighted by Maron et al. (10) in their statement on eligibility for competitive athletes with cardiovascular anomalies. These consensus guidelines create a medical framework for how risk assess young athletes for safe and healthy participation in competitive sports. Some of the topics put forward by these statements include criteria on eligibility and disqualification from organized competitive sports. Of course, every athlete must be individually risk assessed, but having overarching goals helps to guide the individual provider. These statements remain the hallmark of medical decision making, and as mentioned above, will be forever changing.

One way to prevent commotio cordis would be to protect the chest from high-speed blunt trauma. Although football players currently wear cushioned plastic shoulder pads, a chest plate to disperse a physical impact on the heart may reduce the incidence of commotio cordis. Even more importantly, how can we prevent athletes from being struck by blunt objects in sports that don't even have baseline protection, such as softball and baseball? Although only evaluated in animal models, Kumar et al. (11) identified three chest plates that significantly lowered the risk of VF comparted to blunt trauma without a chest protector. We cannot directly link the results of animal studies to human subjects; however, it would be more pragmatic to accept the data as true and potentially modify uniforms to accommodate chest protection. Further investigation would be required to establish definite answers and guidelines, but it seems reasonable that athletes participating in sporting activities where blunt trauma to the chest may benefit from use of basic chest protection. A chest plate to lighten the blow of a high velocity blunt object should not interfere with performance. Baseball and softball players wear helmets for this very reason, why shouldn't their hearts deserve the same protection? However, the best medication is the one patient will take. Young athletes tend to skip out on equipment that they deem unnecessary. Implementation of protective equipment into sports that have not traditionally required such as chest protection will require some buy-in from athletes everywhere, and most importantly, at every level. The case of Damar Hamlin offers an opportunity to increase public awareness of SCD. Professional athletes are positioned to lead this charge. Now may be the best time to team with professional organizations to increase awareness of screening to assess risk for SCD, advocate having defibrillators at the ready at all sports events, and ensure availability of team members trained in advanced cardiac life support to keep sports safe for all who participate.

The media has recently brought to light the severity of commotio cordis in men, but there needs to be a conscious effort to emphasize that women are at risk too. There has been a growth of professional women's sports teams, and women are not just at risk, but likely at increased risk. Guidelines, risk assessment and stratification should also take into account sex differences between men and women. If chest protection for athletes are adopted, there should be various options which specifically accommodate the anatomical differences between men and women. In attempt to reduce bias when developing or suggesting new protective equipment for athletes, we must consider the individual physiology and anatomy of participants.

## The role of arrhythmias and SCD in women

Historically, the role of SCD in women has been underrecognized. Tompkins et al. analyzed data from the MADIT trials to examine sex differences in mortality and device efficacy in patients that received implantable cardiac defibrillator (ICD) or cardiac resynchronization therapy-defibrillator (CRT-D) implantation (1). Overall, the investigators found that death from an arrhythmogenic cause was similar between men and women following ICD or CRT-D implantation and not the main driver of mortality. Interestingly, they found that CRT-D decreases both all cause and cardiac mortality more in women than men. The findings highlight the important fact that women were found to have a higher all-cause mortality as compared to when in those with non-ischemic cardiomyopathy (NICM).

The role of arrhythmias and cardiac mortality in women both needs to be (1) better studied and (2) better publicized among health care providers. To equally serve female patients, we need to fully understand the underlying cause of the substrate that appears as differences in mortality in patients with NICM. For practitioners, there needs to be an emphasis on adjusting the pre-test probability of underlying differences when thinking about arrhythmogenic SCD in women.

## Future directions

Knowing this information, the future should be to (1) ensure there is more public health information on SCD, implementation of risk assessment and risk stratification of SCD by health care providers that considers biological differences between men and women (2) implementation of new technology to ensure the rates of SCD in athletes for screening and prevention. The use of technology and how to better incorporate devices, techniques, systems, in sports requires interdisciplinary discussion amongst specialists in sports medicine, cardiology and public health. One thought is to leverage machine learning algorithms to develop better screening systems for athletes. Students entering middle school and high school sports events can receive yearly EKGs to help screen for underlying cardiac disease. Wearable devices that are appealing to a young audience, can be used to obtain reliable ECG data for screening, risk stratification and possibly diagnosis. If not now, then when?

Recent events on the football field cause shock and awe that recovery can be swift with timely defibrillation and resuscitation, but it is also a wakeup call that more still needs to be done. What can we do to further minimize the risk of SCD of male and female athletes in sports around the world. It is the responsibility of health care providers to speak up about the importance of keeping the hearts of athletes healthy and well.

## Author contributions

All authors listed have made a substantial, direct, and intellectual contribution to the work and approved it for publication.

## References

[1] Tompkins CM Zareba W Greenberg H Goldstein R McNitt S Polonsky B. Differences in mode of death between men and women receiving implantable cardioverter-defibrillators or cardiac resynchronization therapy in the MADIT trials. Heart Rhythm. (2023) 20:39–45. 10.1016/j.hrthm.2022.08.018 36007729

[2] Zeppenfeld K Tfelt-Hansen J de Riva M Winkel BG Behr ER Blom NA. 2022 ESC guidelines for the management of patients with ventricular arrhythmias and the prevention of sudden cardiac death: developed by the task force for the management of patients with ventricular arrhythmias and the prevention of sudden cardiac death of the European Society of Cardiology (ESC) Endorsed by the Association for European Paediatric and Congenital Cardiology (AEPC). Eur Heart J. (2022) 43:3997–4126. 10.1093/eurheartj/ehac262 36017572

[3] Haukilahti ME Holmström L Vähätalo J Kenttä T Tikkanen J Pakanen L. Sudden cardiac death in women: causes of death, autopsy findings, and electrocardiographic risk markers. Circulation. (2019) 139:1012–21. 10.1161/CIRCULATIONAHA.118.037702 30779638

[4] Waase MP Mutharasan RK Whang W DiTullio MR DiFiori JP Callahan L. Electrocardiographic findings in national basketball association athletes. JAMA Cardiol. (2018) 3:69–74. 10.1001/jamacardio.2017.4572 29214319 PMC5833528

[5] Maron BJ Estes IIINM. Commotio cordis. New Engl J Med. (2010) 362:917–27. 10.1056/NEJMra0910111 20220186

[6] Westreich R Haim M Bereza S Konstantino Y. Commotio cordis: indeed? JACC Case Rep. (2019) 1:597–601. 10.1016/j.jaccas.2019.09.010 34316887 PMC8289088

[7] Corrado D Pelliccia A Bjørnstad HH Vanhees L Biffi A Borjesson M. Cardiovascular pre-participation screening of young competitive athletes for prevention of sudden death: proposal for a common European protocol: consensus statement of the Study Group of Sport Cardiology of the Working Group of Cardiac Rehabilitation and Exercise Physiology and the Working Group of Myocardial and Pericardial Diseases of the European Society of Cardiology. Eur Heart J. (2005) 26:516–24. 10.1093/eurheartj/ehi108 15689345

[8] Bickel T Gunasekaran P Murtaza G Gopinathannair R Gunda S Lakkireddy D. Sudden cardiac death in famous athletes, lessons learned, heterogeneity in expert recommendations and pitfalls of contemporary screening strategies. J Atr Fibrillation. (2019) 12:2193. 10.4022/jafib.2193 32435342 PMC7237076

[9] Maron BJ Mitten MJ Quandt EF Zipes DP. Competitive athletes with cardiovascular disease-the case of Nicholas Knapp. New Engl J Med. (1998) 339:1632–5. 10.1056/NEJM199811263392211 9828254

[10] Maron BJ Zipes DP Kovacs RJ. Eligibility and disqualification recommendations for competitive athletes with cardiovascular abnormalities: preamble, principles, and general considerations: a scientific statement from the American Heart Association and American College of Cardiology. Circulation. (2015) 132:e256–61. 10.1161/CIR.0000000000000236 26621642

[11] Kumar K Mandleywala SN Gannon MP Estes IIINA Weinstock J Link MS. Development of a chest wall protector effective in preventing sudden cardiac death by chest wall impact (commotio cordis). Clin J Sport Med. (2017) 27:26. 10.1097/JSM.0000000000000297 27014942 PMC5181132

